# Cancer genomic data systems in precision oncology: from integration to clinical implementation

**DOI:** 10.1016/j.ebiom.2026.106480

**Published:** 2026-09-25

**Authors:** David Torrents, Oliver Hofmann, Karen Ann Cranston, Raffaella Casolino, Andrew V. Biankin, Andrés F. Cardona, Robert L. Grossman, Amber Johns, Mark Lawler, Kjong-Van Lehmann, Raquel Manzano Garcia, Nicola Mulder, Travis Osterman, Timothy Rebbeck, Solomon Rotimi, Zbyslaw Sondka, Shawn Sweeney, Samuel Volchenboum, Alex Wagner, Jianmin Wu, Takayuki Yoshino, Mélanie Courtot, Lincoln Stein

**Affiliations:** a Life Sciences Department, Barcelona Supercomputing Center (BSC), Barcelona, Spain; b Institució Catalana per la Recerca i Estudis Avançats (ICREA), Barcelona, Spain; c University of Melbourne, Melbourne, Australia; d Techna Institute, University Health Network, Toronto, Canada; e International Cancer Genome Consortium, University of Glasgow, Glasgow, United Kingdom; f Direction of Research and Education Luis Carlos Sarmiento Angulo Cancer Treatment and Research Center (CTIC), Bogotá, Colombia; g Department of Medicine and Computer Science, University of Chicago, Chicago, IL, USA; h Center for Translational Data Science, University of Chicago, Chicago, IL, USA; i Queens University Belfast, Belfast, United Kingdom; j Department of Biology, RWTH Aachen University, Aachen, Germany; k Joint Research Center for Computational Life Sciences, Uniklinik RWTH Aachen, Aachen, Germany; l Center for Computational Life Sciences, RWTH Aachen University, Aachen, Germany; m Cancer Research UK Cambridge Institute, Cambridge, United Kingdom; n Computational Biology Division, IDM, University of Cape Town, Cape Town, South Africa; o Vanderbilt University Medical Center, Nashville, TN, USA; p Dana-Farber Cancer Institute, Boston, MA, USA; q Department of Biochemistry, Covenant University, Ota, Nigeria; r COSMIC, Wellcome Sanger Institute, Cambridge, UK; s AACR Project GENIE Coordinating Center at the American Association for Cancer Research, Philadelphia, PA, USA; t Department of Pediatrics, University of Chicago, Chicago, IL, USA; u Institute for Genomic Medicine, Nationwide Children's Hospital; Department of Pediatrics, Ohio State University, Columbus, OH, USA; v State Key Laboratory of Holistic Integrative Management of Gastrointestinal Cancers, Beijing Key Laboratory of Carcinogenesis and Translational Research, Center for Cancer Bioinformatics, Peking University Cancer Hospital & Institute, Beijing, 100142, China; w Peking University International Cancer Institute, Peking University, Beijing, 100191, China; x Department of Global Oncology, Division for Promotion of Drug and Diagnostic Development, Department of Gastrointestinal Oncology, National Cancer Center Hospital East, Kashiwa, Japan; y Genome Informatics, Ontario Institute for Cancer Research, Toronto, Canada; z Medical BioPhysics Department, University of Toronto, Toronto, Canada; aa Department of Computer Science, University of Toronto, Toronto, Canada; ab Ontario Institute for Cancer Research, Toronto, Canada; ac Department of Molecular Genetics, University of Toronto, Toronto, Canada; ad Vector Institute for Artificial Intelligence, Toronto, Canada

**Keywords:** Oncology, Data sharing, Data policy, Data management, Genomics platforms, International governance

## Abstract

Cancer genomic data sharing is a core pillar of precision oncology, underpinning reproducibility, scalability, and equity. The ability to discover, access, integrate, interpret, and reuse cancer ‘omics data is a key determinant of precision oncology's value and benefit at scale. This work has been conducted as part of *The Lancet Oncology* Commission on Cancer Genomics and Precision Oncology to examine the practical data architecture required to support cancer genomics in research, clinical care, and health-system learning. We conclude that without robust data stewardship, including effective data sharing and global integration, the potential of cancer genomics will remain fragmented across projects, platforms, and jurisdictions. This disaggregation limits reproducibility, equity, patient benefit, and societal value. Healthcare systems must prioritise implementing fully efficient data ecosystems as a central challenge for precision oncology.

## Introduction

Cancer genomics and precision oncology are fundamentally dependent on integrating diverse, complex data. Molecular profiles, clinical characteristics, treatment exposures, imaging, pathology, and outcomes must be brought together to generate meaningful insights, guide individual patient care, and enable cumulative learning across populations.^1^ In this context, data integration is a central pillar of precision oncology: without the ability to connect and interpret data across sources, the promise of genomic medicine–matching the right patient to the right intervention at the right time–cannot be realised.

Despite major advances in our ability to generate data at an ever-increasing pace and volume, the actual integration of cancer genomic and clinical data remains limited in practice. Currently, cancer genomics data are generated in different settings for diverse purposes. In the research context, cancer researchers usually generate comprehensive molecular profiles, typically including exome or whole genome sequencing, coupled with rich clinical data to enable new discoveries. On the other hand, in routine clinical practice, narrow targeted gene panels are employed to support diagnosis, prognosis, and treatment selection. However, both settings pose similar challenges. Datasets are often fragmented across institutions, platforms, and jurisdictions; structured using inconsistent standards, and governed by heterogeneous access frameworks. These limitations impede the aggregation of evidence, reduce comparability across studies, and constrain the reproducibility of analyses. As a result, the knowledge needed to interpret variants (particularly rare ones), understand tumour heterogeneity, and optimise treatment strategies accumulates slowly and unevenly. Although only a small fraction of variants can be currently translated into therapeutic decisions, the systematic and consistent integration of genomic and clinical data will expand their clinical applicability to additional genes and tumour types. This fragmentation also risks exacerbating global inequities, as the capacity to integrate and reuse data remains concentrated in a small number of well-resourced settings.

*The Lancet Oncology* Commission on Cancer Genomics and Precision Oncology (hereafter referred to as TLO Cancer ‘Omics Commission) was established to address a central challenge for contemporary cancer care: how to translate rapid advances in genomics and molecularly informed treatment into implementation strategies that are evidence-based, equitable, and sustainable across diverse health-system settings. Although much of the early development of precision oncology has been driven by technological innovation and proof-of-concept studies, the greatest challenges in this field are represented by governance, delivery models, regulatory alignment, workforce capacity, and data integration. Central to TLO Cancer ‘Omics Commission findings and recommendations is the need for health systems to move beyond isolated data silos towards integrated, learning data ecosystems that can support continuous improvement in care. Such systems must enable data to flow responsibly across institutional and national boundaries, while maintaining trust, protecting confidentiality, and ensuring that benefits are shared equitably (Panel 1).

Panel 1 **Principles for cancer genomic data stewardship in the context of the Lancet Oncology Commission on cancer genomics and precision oncology.**

*The Lancet Oncology* Commission on cancer genomics and precision oncology frames precision oncology as a system-level transformation. This framing has important implications for cancer genomic data systems. The vision set out by the Commission rests on several interconnected principles:

• **Integration for clinical utility:** Molecular findings derive value from comparison across datasets and linkage to treatment and outcome data. Data ecosystems must therefore enable integration across sources and settings.
• **Equity through representation and participation:** Precision oncology cannot be equitable if data resources are incomplete, non-representative, or concentrated in a small number of institutions and countries. Data stewardship should support broader participation in both data generation and data use.
• **Trust through governance and security:** Responsible reuse of cancer genomic data depends on transparent governance, proportionate data protection, and secure access mechanisms that sustain confidence among patients, clinicians, institutions, and regulators.
• **Sustainability:** Precision oncology cannot depend on fragile, short-term, project-based repositories. Durable data infrastructures, stable financing, and long-term stewardship are essential for cumulative learning and continued value.

These principles underscore that data architecture is integral to the implementation of precision oncology.

This technical paper complements the TLO Cancer ‘Omics Commission by expanding on the data architecture required to enable such integration. It describes how technical standards underpin the effective use of data for clinical decision-making, research, and system learning. We argue that the principal barriers to precision oncology are increasingly located not only in data generation or interpretation, but in the implementation of data ecosystems that are findable, accessible, interoperable, and reusable (FAIR),^2^ while also being secure, governed, and sustainable. Achieving meaningful data integration therefore requires a concrete shift from aspirational data sharing to active data stewardship. Such stewardship is essential to ensure that cancer genomic data can serve not as isolated outputs of individual projects, but as enduring assets for clinical decision-making, research, and health-system learning. Through this technical guidance, we provide an operational perspective on one of the key pillars underpinning the Commission's vision and outline the practical data architecture needed to support precision oncology at scale.

## The guiding principles of research data sharing: FAIR

For nearly a decade, the genomic research community has committed to principles of data sharing known as FAIR—an acronym that stands for *Findable, Accessible, Interoperable,* and *Reusable*. These principles apply to all types of data and are designed to promote responsible stewardship of large data sets and maximise their long-term utility to the community. All current efforts to design and implement digital infrastructures for managing oncology data are guided by FAIR. Achieving interoperable and reusable datasets requires the implementation of FAIR principles across the entire data lifecycle, from data acquisition to long-term reuse in both research and healthcare settings (Fig. 1).

**Fig. 1:**
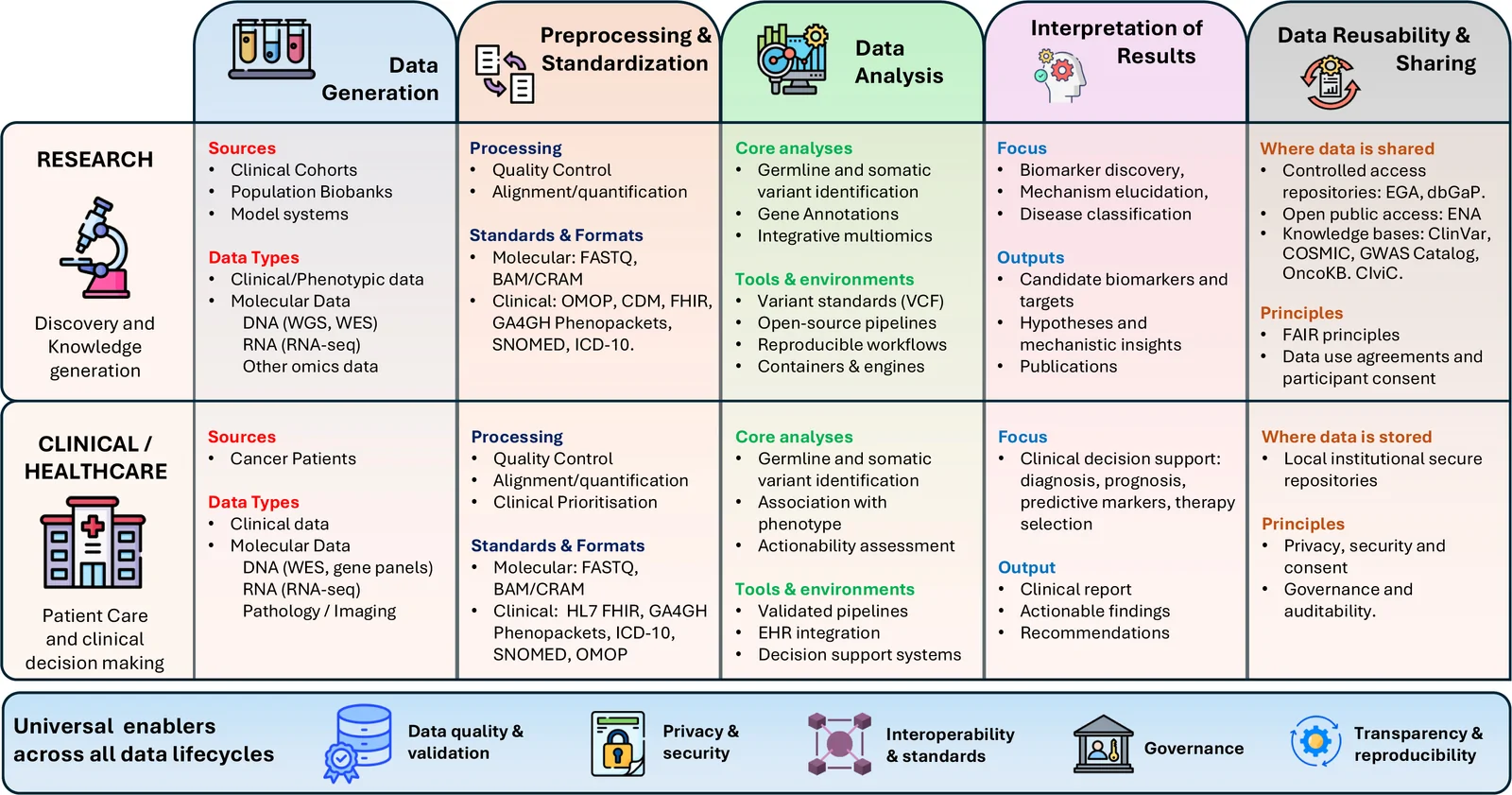
**Implementation guidance across the oncology genomic and clinical data lifecycle in research and healthcare.** The oncology genomic and clinical data lifecycle comprises five interconnected phases: (1) data generation, (2) data preprocessing and standardisation, (3) data analysis, (4) interpretation of results, and (5) data reusability. Although these phases are common to both research and healthcare, their implementation differs according to the ultimate use of the data. Whereas research prioritises maximising discovery and secondary use through wide data collection, methodological flexibility, and reproducibility, healthcare settings are focused on supporting patient care by maximising validated data generation, analytical robustness, and regulatory compliance. For data processing and standardisation, both settings seek to maximise interoperability through common standards, such as FHIR, OMOP, mCODE, Phenopackets for clinical data and FASTQ, BAM and Variant Call Format (VCF) for genomic data, with healthcare requiring, in this case, additional validation and traceability. Data analysis in research prioritises reproducible and flexible computational workflows, while healthcare prefers validated analytical pipelines that maximise precision. The Interpretation of results differs substantially between both settings. Research uses, for example, frameworks from the Variant Interpretation for Cancer Consortium (VICC), Cancer Genomics Consortium (CGC), and the ClinGen Somatic Working Group, complemented by standardised variant identifiers from the ClinGen Allele Registry and public knowledgebases, such as ClinVar, CIViC, and OncoKB to study variant oncogenicity and tumour biology. In contrast, healthcare interpretation tends to rely on the AMP/ASCO/CAP guidelines for somatic variant classification, complemented with OncoKB, ClinVar, and CIViC to inform diagnostic, prognostic, and therapeutic decisions. Community surveys^3^ revealed, for example, that many laboratories customised aspects of the AMP/ASCO/CAP framework during implementation (e.g., evidence weighting, use of internal knowledge bases, and reporting thresholds), further contributing to overall heterogeneity for variant interpretation. Beyond the reuse of interpreted results through public knowledgebases, access to raw genomic and clinical data is essential for both settings. Whereas research promotes controlled data sharing through repositories such as EGA, dbGaP, and ENA, the reuse of raw data in healthcare is generally more restricted, often limited to local use due to governance, privacy, and regulatory requirements.

### Finding the data

#### Database portals

A fundamental requirement for data to be shareable and reusable within the community is that it must first be discoverable within data repositories. For example, a cancer researcher studying uncommon mutations in the *BRCA2* gene might wish to create a collection of genomic and clinical data from all patients with mutations in this gene. To make these data findable, the repository must systematically annotate each patient record with the list of mutated genes and index the mutations for rapid retrieval. This is generally done using a database management system. Then, for the researcher's convenience, the repository should provide a user-friendly “portal” or gateway that enables the researcher to execute this and other searches. In the interests of making the dataset visible to other researchers, data deposition in a well-known public repository, such as the European Genome-Phenome Archive (EGA)^4^ or the database of Genotypes and Phenotypes (dbGaP),^5^ is recommended.

#### APIs

A search portal alone is not adequate. What if there are several repositories containing similar data, and a researcher wishes to search all of them for records of patients with a similar *BRCA2* mutation? Or what if the researcher has written software that needs to access the repository unattended? To make this possible, it is strongly recommended that cancer data repositories provide a published Application Programming Interface (API) that enables software developers to write scripts and larger applications to search the database automatically.^6^

#### Federated systems

Increasingly, cancer data cohorts are published by multiple groups, sometimes as part of large consortia, and at other times independently. To perform a cross-cohort analysis, researchers have to combine data from multiple datasets. The traditional way to enable this is to merge the cohorts into a single central database (Fig. 2A). This is the approach taken by the large Genomic Data Commons (GDC^7^) and European Genome/Phenome Archive (EGA^8^) repositories.

**Fig. 2:**
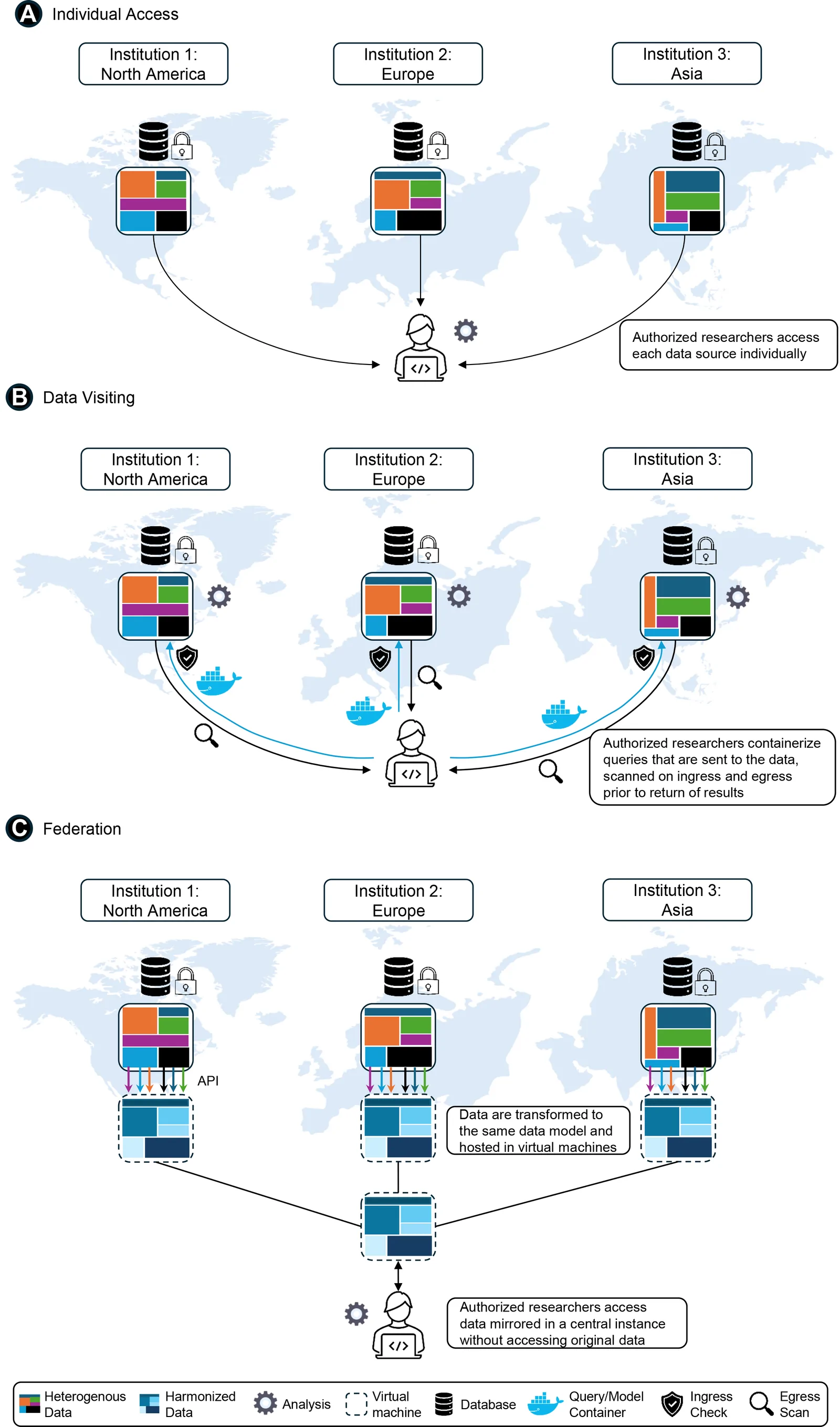
**Paradigms for data sharing. A**. Independent institutional repositories maintain cancer data across diverse jurisdictions, protected by local firewalls. These datasets can be stored using heterogeneous schemas; data users get permission and access each repository independently to analyse the data locally. **B**. The “Data Visiting” model leverages software containers to transmit analytic models or queries directly to the data repository. Ingress scanning ensures security against malicious software, while egress monitoring prevents the unauthorised removal of protected health information. Additional restrictions, such as disabling internet access within the environment, can further mitigate data leakage risks. **C**. The Federated model is a decentralised organisation, where data remains stored at its original source. Researchers can pull preprocessed (aggregated) or processed data and results directly from different locations through a centralised interface. Interoperability is achieved by transforming datasets into community-supported common data models. Harmonised data are transformed and integrated via application programming interfaces into virtual machines situated behind institutional firewalls. This architecture enables a unified virtual representation for researchers while allowing individual institutions to maintain granular control over which data are mirrored. Similar frameworks can be utilised to support the federated training of large language models.

However, for practical and legal reasons, it may not be possible to combine cancer cohorts in a single location, in which case the cohorts will reside in separate databases. To perform cross-cohort analysis, a cancer researcher must run the desired search on one database, retrieve the results, repeat the search on the second database, merge the results with the first, and so forth. In general, each existing repository of cancer ‘omics data has a different search interface, forcing researchers to search each one manually, or to write custom code to do it.

The recommended solution to this problem is to adopt a standard search API such as the Beacon protocol^9^^,^^10^ developed by the Global Alliance for Genomics in Health (GA4GH). This protocol provides a standardised way to determine whether a human genome database contains records for patients that match a variety of common criteria, including the presence of a germline or tumour variant, sex, age, and cancer type. The Beacon also provides a lightweight form of data federation by allowing a researcher to simultaneously search for cancers with a particular variant across multiple databases.

### Accessing the data

Finding the desired cancer data is only the first step. Once a researcher identifies the desired data in a shared repository, they need to access its contents to enable analysis. There are two technical challenges here. The first is the mechanism for accessing the contents of a data object for analysis. The second is interpreting the data contents.

Traditionally, cancer researchers who needed to work with shared ‘omic-scale datasets would download the data to their local systems from an online repository, making a local copy. If they needed to combine data from two repositories, they would download from each repository separately and merge the results (Fig. 2A). This mechanism, while still widely used, has several drawbacks. First, downloading large datasets is time-consuming and wasteful of resources, as there end up being local copies of the same dataset in multiple locations around the world. Second, once a dataset has been copied, it is difficult to control how it is used, how securely it is handled, and whether it is being redistributed. This becomes an ethical and legal issue when the data contain potentially identifiable information.

A second emerging data access method is known as “Data Visiting” (Fig. 2B), also known as “secure research environments”. Here the cancer data are stored online in a cloud computing service that provides both data storage and the compute resources needed to analyse them. The researcher is not allowed to download the data but instead logs in to the cloud system and performs the desired analysis within the environment. This has several advantages. The researcher can get to the cancer data quickly without a time-consuming download step. They can also leverage the substantial computational resources offered by cloud environments without investing in significant local hardware. For the data provider, data visiting ensures that data are analysed in a secure environment, enables logging and monitoring services, and helps ensure that data are used in a manner consistent with the participants’ consent. A prime example of this approach is the Genomics England Research Environment (RE),^11^ a secure, cloud-based platform designed for approved researchers to analyse data from over 100,000 sequenced genomes.

One downside of the Data Visiting paradigm is that it can be inconvenient for cancer researchers to set up their preferred analytic environment in the data provider's cloud, and cost can become quite high for large volumes of data. However, a more serious problem is that combining information from two providers' datasets requires setting up two separate analytic pipelines to accommodate the idiosyncrasies of each cloud environment, and then developing code to merge the outputs of those pipelines. There is also the challenge that some legal jurisdictions prohibit storing cancer omics data in a cloud computing environment. An emerging practice is for two, sometimes more, cloud environments to interoperate in a secure and compliant manner, enabling researchers in one cloud environment to access, analyse, and integrate data from the other cloud environment.^12^ Sometimes this is viewed as “extending the boundary” of one cloud environment to encompass another.

In practice, the current way of accessing and combining shared cancer ‘omics datasets is an *ad-hoc* hybrid of the two methods (Fig. 2C), called “Federated Data.” Some datasets are accessible only by visiting, others by downloading, and some of the analysis is performed in the cloud, while other parts are run on local resources.

### Making oncology data interoperable

Consider three cancer datasets that contain information on several tumour types, including breast cancer. In one dataset breast tumours are referred to as “BRCA”. In the second, these tumours are named “invasive breast cancer,” and in the third, “invasive ductal adenocarcinoma.” Are these three terms referring to the same histopathological entity, or are there differences among them? The “I” in FAIR stands for “interoperable.” In the context of cancer data sharing, this refers to the ease with which two or more datasets produced by independent groups can be merged for joint or comparative analysis.

Cancer repositories are strongly encouraged to use widely accepted, community-supported controlled vocabularies, data models, and file formats whenever possible. Controlled vocabularies are machine- or human-readable codes with explicitly defined meanings. For example, the Oncotree^13^ controlled vocabulary assigns the code “IDC” to Breast Invasive Ductal Carcinoma and links it to a detailed description of the disease.

In addition to using well-known controlled vocabularies, repositories are encouraged to use shared data models. Data models typically include several controlled vocabularies, but go beyond them by describing how the fields in a data record relate to each other.

### Making oncology data reusable

Reusability, the final pillar of FAIR, ensures that cancer data collected through the generosity of patient donors and the hard work of many clinicians and researchers continue to provide value to the research and healthcare communities. The ability to reuse data and results across diverse oncological studies significantly enhances cancer research. This can be achieved through more powerful integrative analyses of existing and new datasets, such as those from the same tumour type, or by reanalysing original data using improved, more advanced analytical methods, such as Artificial Intelligence (AI). Early projects like TCGA and ICGC exemplify collaborative environments where raw and processed data are shared and reused for both discovery and benchmarking, and the ongoing impact of these data-sharing efforts is still felt. Although the TCGA consortium dissolved in 2019, a PubMed search for “cancer TCGA” still identified more than 5000 manuscripts published in 2024. It is crucial to design and implement data infrastructures that enable efficient interoperability, discovery, and access to oncological data; this includes keeping archives of raw cancer data whenever feasible.

Data reusability becomes critically important as our understanding of biology progresses rapidly. For example, a major clinical challenge is interpreting a novel genomic variant observed in a patient's genome. Is it a “passenger” mutation that has no functional significance, or is it a pathogenic variant that has significance for the course of the patient's disease? By maintaining large globally accessible repositories of such “variants of uncertain significance” and their clinical correlates, and by allowing them to be reused for long after the initial study or diagnostic test that identified them, the community has been able to build standards and services that provide clinicians with the tools to distinguish among pathogenic and non-pathogenic variants.^3^^,^^14^, ^15^, ^16^, ^17^, ^18^

Despite this progress, much remains to be done to ensure data can be reused effectively. For example, when developing a consensus knowledgebase of oncogenic variants in haematological disorders, the American Society of Haematology Somatic Working Group was unable to resolve conflicting classifications across submitting institutions.^19^ This challenge was primarily attributed to a lack of computable evidence and provenance underlying the submitted variant interpretations. Reusability of cancer variant knowledge is also hampered by a lack of data standards for the representation of aggregative classes of “categorical variants”^20^, ^21^, ^22^ used in these resources, such as “EGFR exon 19 skipping” and “ALK oncogenic mutations”. While such categorical terms are widely used and offer practical value in clinical workflows, they can obscure clinically meaningful differences between the individual variants they group together. For example, distinct ALK resistance mutations show differential sensitivity to structurally different ALK inhibitors, meaning the specific variant present should inform treatment choice.^23^ Data standards to address both challenges are under development within the Genomic Knowledge Standards Work Stream of the GA4GH.^24^

### Making analytic research workflows FAIR

FAIR principles also apply to data analysis software and the workflows that describe how a series of analytic steps are strung together. Data analysis has become a fundamental pillar of cancer genomic research, yet challenges persist in standardising and managing workflows across research and clinical settings. Reproducibility remains a major issue: published evaluations report very low and heterogeneous reproducibility rates, roughly 0–26%, with the review estimating around 20% overall,^25^ highlighting the technical barriers to computational reproducibility across the field.

The analytic workflow equivalent of FAIR is FAIR4RS (FAIR for Research Software).^26^^,^^27^ FAIR4RS adds to FAIR the requirement that data should not only be accessible and reusable by individuals, but also be explicitly useable by automated tools, for example through published APIs. However, one practical challenge for tool developers is the rapidly evolving nature of software and data ecosystems, in which software libraries, tools, and the reference databases that underpin these workflows are continually changing. An additional complication is the sheer complexity of cancer data analysis. The many tunable steps involved in moving from raw signal files to biologically relevant variants necessitate different specialised tools for each step,^25^, ^26^, ^27^ and each step is continually evolving as improvements are made to enhance accuracy and efficiency.

To mitigate these concerns, we strongly recommend using formal workflow management systems such as Nextflow, Snakemake, or WDL (Workflow Description Language), which provide a high-level description of the analytic steps required to perform a desired task and promote modularity and reusability.^28^ Similarly, we recommend that analytic software development groups apply automated validation tools to ensure that the data files produced by their software are standards-compliant. To address the challenges of installing and configuring complex software packages, we recommend using software containers, such as Docker, which bundle software, configuration files, and dependencies into a single portable package.^29^

## Technical means to protect patient confidentiality

In tension with the goal of promoting FAIR access to cancer data are the ethical and legal obligations to protect the confidentiality of patients with cancer who have donated their information. Informed consent agreements invariably contain terms that commit the research groups that receive and generate the data to protect donors' identities. While directly identifying information, such as the donor's name, address, or medical record number, can be removed from the repository, there is always a non-zero probability of inferring the donor's identity by combining core clinical information (disease type, treatment, hospital location) or by using germline variants in the tumour genome to interrogate genealogical databases for matches to the donor or their relatives. Datasets that include germline sequencing information are much more easily identifiable than those that include only somatic mutations and require additional security measures. Because somatic mutations are changes specific to the patient's cancer, they cannot be used to identify the patient unless the attacker gains access to the tumour tissue itself.

The primary means of preventing users of the data from attempting to re-identify donors is through legal agreements: data use agreements between data custodians and researchers who wish to access it. Here, we discuss additional technical measures to reduce the likelihood of deliberate or inadvertent violations of confidentiality.

### Best security practices for cancer data repositories

Cancer research data are vulnerable to interception by unauthorised third parties when they are transmitted across the internet and when they are stored in a file system or database. Information can be compromised, held for ransom, exfiltrated by bad actors or inadvertently leaked by legitimate users. At the very least, cancer data should be stored online in accordance with the current best practices for IT security, which include encryption of the data, the use of multi-factor authentication, and the implementation of data access monitoring systems.^30^ Additional IT security recommendations specific to repositories that provide access to cancer and other personal health information can be found in these publications.^31^^,^^32^

## Securing Shared Cancer Research Data

As cancer data analysis becomes increasingly reliant on integrating data from multiple repositories, security issues become more complex. Referring to Fig. 2, recall that there are three general models for data sharing and integration. The traditional model, Independent Databases (Fig. 2A), presents the greatest challenges to protecting confidentiality because cancer data are physically copied from the data repository to the local computer system or cloud service hosted by the researcher. Once the data are copied, their security depends on the strength of the local system's configuration and may be vulnerable to unauthorised access. In addition, once the data have been copied to a third-party site, there is no technical means to prevent them from being copied successively to other locations.

In this scenario, the data custodians rely on legal agreements, such as attestations from researchers' host institutions, that the researchers will comply with data-use restrictions, destroy their copies of the data once their research has concluded, and that the local computer systems implement recommended cybersecurity protocols. Data access logging is used to monitor who has accessed the data in order to enable forensic analysis of a data breach.

In contrast, the Data Visiting^33^ model (Fig. 2B) is generally considered to offer greater control over how cancer data are accessed, which can enhance patient confidentiality protection under appropriate governance. However, it can restrict researchers' ability to integrate different datasets, as noted earlier. The Federated model (Fig. 2C) is a hybrid of the first two architectures; its data–security profile relative to the other models depends heavily on implementation details, governance arrangements, the specific threat model considered, and the particular federation architecture adopted, rather than being an inherent property of the approach itself.

It is also worth noting that publicly reported incidents of confidential cancer patient data being disclosed from a research repository appear to be uncommon, although the true incidence is difficult to establish given inconsistent reporting requirements across jurisdictions and the likelihood that some breaches go undetected or unpublished. This should not be taken to imply that any one architecture is inherently secure; robust governance, technical safeguards, and monitoring remain essential regardless of the data-access model used.

### Sustainability and reliability

When cancer genomic data are FAIR, they can be reused, repurposed, and combined in innovative and productive ways. Advances in discovery research and technologies such as Artificial Intelligence/Machine Learning (AI/ML) are allowing us to capture increasingly complex signals of tumour biological processes from these legacy datasets. The data collected today will still be relevant a decade from now. Indeed, it may increase in value over time.

For this reason, every large-scale cancer genomic research project must have a plan for ongoing long-term maintenance of the data resource. To ensure the long-term sustainability of a data repository, three critical elements must be in place. Firstly, stable funding is essential to support ongoing operation of the repository infrastructure. Secondly, the resource must maintain the staffing necessary to collect, integrate, and distribute new data. Lastly, the repository must maintain consistent relevance to its end users, for example by supporting new analytic tools and techniques. Because of the costs and expertise required to operate a data repository sustainably, it is generally recommended to use government-supported repositories designed to manage data from multiple projects. Sometimes, however, there is no suitable repository for a particular dataset, in which case a new repository must be designed with sustainability in mind.

### Disaster preparedness

The combination of high operating costs and the custody of high-value, irreplaceable datasets make oncology data repositories uniquely vulnerable to loss of funding or catastrophic events such as a ransomware attack. Because of risks like these, we strongly recommend that every cancer repository have a disaster preparedness plan in place and be prepared to execute it. Such a plan must, at a minimum, ensure that the data are backed up to location(s) distant from the primary storage site, that there are archived copies of the software needed to operate the repository, and that the protocols for operating the repository, such as curator training materials and system administration manuals, are backed up and accessible as well. The goal is to ensure that after a catastrophic event, whether a cyberattack, a fire, or loss of funding, the resource can eventually be restored to operational status–though it often may not fully recover.

There are multiple mechanisms to achieve this aim, including backing up the cancer data to a reliable commercial cloud storage system, creating virtual images of the servers that run the repository's website, backend database, and analysis tools, or creating mirror sites in geographically distant regions. The most appropriate solution depends on the repository's particular circumstances. Two recently established independent certification agencies can help vet a repository's disaster preparation plans. The Global Biodata Coalition^34^ is a consortium of biological research funding agencies that seeks to enhance equitable access to biological and biomedical data. Among the services it provides is an accreditation system for essential (“core”) biomedical data repositories. Repositories that receive the Global Core Biodata Resource designation have met multiple stringent requirements, including requirements related to sustainability and disaster preparedness.

Similarly, the CoreTrustSeal, an accreditation service provided by the CoreTrustSeal Foundation,^35^ certifies that a digital data repository meets multiple requirements for being a trustworthy repository. These include a well-defined data governance structure, reliable, traceable data management, security provisions, quality control, and a robust plan for service continuity. Several key resources for cancer omics, including the UniProt^36^ repository of protein sequences, the Reactome^37^ knowledgebase of reactions and pathways, and CNSA,^38^ a Chinese repository of multiple cancer and non-cancer genomic data sets, have all recently been recognised with this seal.

We recommend that cancer data repositories obtain certification from one or both of these entities, and that cancer researchers encourage the repositories they depend on to do so as well.

## Outstanding questions

Important questions remain about how cancer genomic data ecosystems can be implemented at scale while preserving trust, equity, and long-term value. As data volumes increase across multiple platforms–including genomics, pathology, imaging, registries, clinical records, and research databases–future research should examine how these sources can be linked and integrated in ways that are technically robust, clinically meaningful, and feasible across health systems. Greater attention is also needed to define the role of artificial intelligence, including Large Language Models, in curating, harmonising, analysing, and interpreting these increasingly complex data streams, while ensuring transparency, reproducibility, and appropriate oversight. At the same time, research must address the growing risks to patient confidentiality associated with linkage across datasets and jurisdictions, and identify governance and technical safeguards that enable responsible use. Finally, considerations around the data lifecycle–from the sample provided by the human participant to the impact on new discoveries and treatments–remain unanswered: how can we provide greater transparency in usage to all communities, from the patients who provided the original consent, the data access committee that provided the data to the researcher, to the study that leveraged these data and produces new results.

## Conclusion

Advancing precision oncology depends on a collective commitment to open, well-governed cancer genomic data sharing. **Policymakers** must recognise data sharing as central to the field's progress, ensuring sustained funding for training data professionals and for the infrastructure needed to maintain accessible, high-quality resources. Besides funding, clear accountability frameworks should assign responsibility for data maintenance and adherence to FAIR principles across individual researchers, host institutions, and funders alike. Without these structural foundations, even the most promising genomic discoveries will remain siloed, limiting their translational impact to human health.

Researchers, institutions, and repository managers each carry distinct but complementary obligations in delivering this vision. **Researchers and their institutions** must treat data sharing not as optional but as a core scientific duty, embedded from the conceptual stage of their projects, and commit to FAIR principles and enforcing rigorous data management practices. **Repository managers**, in turn, bear responsibility for long-term sustainability planning, adopting standards-based vocabularies and data models that enable interoperability, and seeking accreditation from recognised bodies such as the Global Biodata Coalition and CoreTrustSeal. Only through this coordinated, multi-stakeholder effort can the cancer genomics community build the trusted, interoperable data ecosystem that precision oncology demands.

**Search strategy and selection criteria**

We identified literature relevant to cancer genomic data systems through searches of MEDLINE, Current Contents, and PubMed, supplemented by hand-searching the reference lists of relevant articles. Search terms included combinations of “cancer genomics”, “precision oncology”, “omics”, “data sharing”, “data stewardship”, “FAIR”, “interoperability”, “federated data”, “data governance”, “security”, “repository”, “workflow reproducibility”, “variant interpretation”, and “sustainability”. Abstracts and reports from scientific meetings were included only when they related directly to previously published work. Only articles published in English between Jan 1, 2001, and Dec 31, 2025, were included. Articles were selected for relevance to the technical, operational, and governance dimensions of cancer genomic data sharing and reuse. This Review was not designed as a formal systematic review, but aimed to capture key conceptual, technical, and implementation literature relevant to cancer genomics and precision oncology.

## Contributors

MC, RLG, KVL, TO, and LS conceived the concept. AJ and SR performed data curation. AFC, ML, SR, JW, MC, and LS conducted the investigation. RLG, AJ, MC, and LS developed the methodology. AFC and AJ performed formal analysis. AJ, MC, and LS handled project administration, with MC and LS providing resources. AJ, ML, MC, and LS supervised the work. DT, OH, KAC, RC, AVB, AFC, RLG, AJ, KVL, TR, SR, ZS, SS, SV, AW, JW, MC, and LS draughted the original manuscript. DT, OH, KAC, RC, AVB, AFC, RLG, AJ, ML, KVL, RMG, NM, TO, TR, SS, SV, AW, TY, MC, and LS reviewed and edited the manuscript. All authors reviewed and approved the final manuscript.

## Declaration of interests

OH declares grants from Australian BioCommons/NCRIS (GUARDIANS National Genomics Infrastructure) and the US Department of Defence (Breast Cancer Atlas), and membership of the GA4GH Strategic Leadership Advisory Board and the ELIXIR GDI Infrastructure Advisory Board. AVB declares grants paid to his institution (University of Glasgow) from Cancer Research UK (ICGC-ARGO; Precision-Panc; Glasgow Centre), Bristol Myers Squibb (Primus 001/002 clinical trials), and the International Cancer Genome Consortium; institutional royalties/licence income from Agilent Technologies; personal consulting fees from Congenica, Concr, and Cumulus Oncology; personal honoraria for lectures/presentations from the University of Wisconsin, the Italian Institute of Genomic Medicine (Turin), and the Istituto Regina Elena (Rome); travel support from Galapagos; participated on advisory boards for Galapagos, Astellas, and Bristol Myers Squibb; and has stock or stock options in Gabriel Precision Oncology, Cumulus Oncology, and Concr. AFC declares grants or contracts from Roche, Boehringer Ingelheim, Novartis, Foundation Medicine, AstraZeneca, QQF, Roche Diagnostics, Amgen, Bayer, CCF, and Idylla; consulting fees from Roche, Boehringer Ingelheim, Pfizer, Novartis, Celldex, AstraZeneca, Astellas, Amgen, MSD, Merck Serono, EISAI, Janssen, BioNTech, Flatiron Health, Teva Pharma, Takeda, FICMAC, and Servier; honoraria for lectures and presentations from Johnson & Johnson, Roche, Boehringer Ingelheim, Pfizer, Novartis, AstraZeneca, Amgen, Bristol Myers Squibb, MSD, EISAI, Teva, Merck Serono, Takeda, Mirati, and Servier; payment for expert testimony from Roche, Boehringer Ingelheim, Novartis, Bristol Myers Squibb, AbbVie, Celldex, Idylla, Thermo Fisher, Illumina, Amgen, Eli Lilly, Guardant Health, Regeneron, Bayer, Biocartis, Pharma Mar, and Pfizer; support for attending meetings and travel from Roche, Boehringer Ingelheim, Novartis, Bristol Myers Squibb, AbbVie, Celldex, Idylla, Thermo Fisher, Illumina, Amgen, Eli Lilly, Guardant Health, Regeneron, Bayer, Biocartis, and Pfizer; participation on data safety monitoring or advisory boards for Roche, Johnson & Johnson, and AstraZeneca; leadership roles with IASLC and ASCO; and receipt of equipment, materials, or services from Roche, Rochem Biocare, Thermo Fisher, and Illumina. RG declares support for the present manuscript from the Center for Translational Data Science, University of Chicago; leadership roles in the Center for Computational Science Research (CCSR, including its Open Commons Consortium and BLOODPAC divisions), The Fund for Innovation in Cancer Informatics (ICI), and Advancing Cancer Treatment (ACT); and receipt of computing credits from Amazon Web Services. AJ declares consulting fees from the University of Glasgow. ML declares honoraria from Pfizer for a presentation, unrelated to this research. TO declares grants from the National Cancer Institute (5P30CA068485-29; 5UH3CA260318-05) supporting the present manuscript; further institutional grants or contracts from GE Healthcare, Microsoft, the Melinda Stead Fund, IBM, and the Conquer Cancer Foundation; consulting fees from AstraZeneca, eHealth Technologies, MD Outlook, Biodesix, Medscape, the Dedham Group, MJH Life Sciences, Tempus AI, COTA Healthcare, Flagship Biosciences, GenomOncology, and Outcomes Insights; and ownership of FacultyCoaching.com. SV declares consulting fees from Belay Diagnostics, paid directly to him; travel support for attending various paediatric cancer meetings, unrelated to this manuscript; and a board membership with CRDSA. TY declares grants or contracts paid to his institution from Bristol Myers Squibb, Caris, Chugai, Daiichi Sankyo, Eisai, Exact Sciences, FALCO Biosystems, Medical & Biological Laboratories, Merus, Miyarisan, MSD, Nareta, Nippon Boehringer Ingelheim, Ono Pharmaceutical, Pfizer, Sysmex, Taiho Pharmaceutical, and Takeda Pharmaceutical; personal consulting fees from Sumitomo Corp and Indivumed; and honoraria for lectures/presentations paid to his institution from Chugai Pharma, Ono Pharmaceutical, Pfizer, Sysmex, Taiho Pharmaceutical, and Takeda Pharmaceutical. MC declares salary support from the Ontario Institute for Cancer Research (OICR) for the present manuscript; grants/contracts supporting the National Data Spaces Pilot Program, the Research Software AI Enhancement Program, iMicroSeq (Genome Canada), the CBW-PCGL Data Management Training proposal (Compute Ontario), the Human Cancer Models Initiative (NCI/Leidos), the Pan-Canadian Genome Library, an OICR New Investigator award, the OHCRN Data Coordination Centre, the ICGC-ARGO Data Coordination Centre, the Overture Platform (NIH U24), CoVaRR-Net, the CanCOGeN Virus Genome Sequencing Project, and EUCANCan (CIHR); consulting fees from F. Hoffmann-La Roche; support for attending meetings and travel from F. Hoffmann-La Roche and the NIH; and participation on boards/advisory groups for EVORA, Roche WAYFIND-R, GA4GH, the Digital Research Alliance of Canada researcher council, the eLwazi Open Data Science Platform, EOSC4CANCER, and PHA4GE. LS declares salary support from the Ontario Institute for Cancer Research, his primary employer, funded by the Government of Ontario. DT, KAC, RC, KVL, RMG, NM, TR, SOR, ZS, SMS, AW and JW declare no competing interests.
